# The Digital Clock and Recall and Its Relationships with Standard Assessment of Alzheimer’s Diagnosis: Insights from the UK SBS‐IONA Clinical Study

**DOI:** 10.1002/alz70861_108883

**Published:** 2025-12-23

**Authors:** Claudio Toro‐Serey, Ali Jannati, Alvaro Pascual‐Leone, Craig Ritchie, Sarah Gregory, Sean Tobyne

**Affiliations:** ^1^ Linus Health, Boston, MA USA; ^2^ Harvard Medical School, Boston, MA USA; ^3^ Boston Children's Hospital, Harvard Medical School, Boston, MA USA; ^4^ Linus Health, Waltham, MA USA; ^5^ Hinda and Arthur Marcus Institute for Aging Research and Deanna and Sidney Wolk Center for Memory Health, Hebrew SeniorLife, Boston, MA USA; ^6^ Department of Neurology, Harvard Medical School, Boston, MA USA; ^7^ Scottish Brain Sciences, Edinburgh, Scotland UK

## Abstract

**Background:**

The Digital Clock and Recall (DCR) is an automated, ML‐based, next‐generation version of the Mini‐Cog, composed of the digital clock drawing test (DCTclock) and 3‐word delayed recall to produce a 0–5 score. Its use has been validated in US‐based research and real‐world clinical studies. Here we evaluated the concordant validity of DCR against commonly used cognitive batteries in a UK sample from the SBS‐IONA study, including the NIH Toolbox Cognition Battery (NIHTB‐CB), Telephone Interview for Cognitive Status (TICS), Addenbrooke’s Cognitive Examination (ACE‐R), and Mini‐Mental State Examination (MMSE). We also evaluated the association between DCR and reported diagnosis of Alzheimer’s disease (AD).

**Method:**

We evaluated 465 participants from the Scottish SBS‐IONA study (median age=69 years [SD=8.3], median education=17 years [SD=3.8], 61% female, 7.2% confirmed AD diagnosis). We used baseline results from the NIHTB‐CB (fluid cognition questions, 1‐5), TICS, ACE‐R, and MMSE. We performed Poisson and Logistic regressions (with covariates for age, sex, and education) to test the concordance of cognitive tests with DCR, and associations of cognitive tests with AD diagnosis.

**Result:**

We found concordance of DCR with NIHTB‐CB (Q4: Z=‐3.17, *p* <0.01; Q5: Z=‐2.04, *p* <0.05), TICS (Z=2.45, *p* <0.05), and ACE‐R (Z=2.91, *p* <0.005). The AD group had significantly lower DCR scores (median = 0, 1.68) compared to unimpaired individuals (median=4, SD=1.41; Z=‐5.46, *p* <0.0001). Finally, the only significant predictors of AD were DCR (Z=‐2.28, *p* <0.05), age (Z=0.27, *p* <0.01), and NIHTB‐CB (Q1: Z=3.02; Q3: Z=‐2.54; Q4: Z=‐1.96; all *p* < 0.05).

**Conclusion:**

The DCR has strong correlations with cognitive batteries widely used for the evaluation of individuals with cognitive impairment. We show for the first time in a representative study from the UK that the DCR stands out amongst such cognitive batteries in identifying individuals with AD. The SBS‐IONA study offers a broad set of clinical factors to further understand what specifically underlies these benefits in the future.

